# Lower Motor Neuron Facial Nerve Paralysis Following Recombinant Hepatitis B Vaccine Administration: A Case Report and Literature Review

**DOI:** 10.1002/ccr3.9655

**Published:** 2024-12-10

**Authors:** Raza Gulzar Ghouri, Hamza Naeem, Mirza Rehan Yousaf, Anam Sohail, Waqas Arshad, Abdul Mabood Basil

**Affiliations:** ^1^ Pakistan Kidney and Liver Institute and Research Center Lahore Pakistan; ^2^ King Edward Medical University Lahore Pakistan; ^3^ Spinghar Medical University Kabul Afghanistan

**Keywords:** Bell's palsy, facial nerve palsy, hepatitis B vaccine, neurological complication

## Abstract

The Hepatitis B vaccine's safety profile is considered safe, but sometimes neurological complications, like Bell's palsy (acute peripheral facial neuropathy), can occur after its administration. A 35‐year‐old female doctor experienced left‐sided facial weakness and paralysis six days after getting the Hepatitis B vaccine. On examination, she had lower motor neuron facial nerve palsy. After excluding other causes, a diagnosis of Bell's palsy was made following Hepatitis B vaccine. She was treated with corticosteroids, antiviral medication, and physiotherapy, which led to a complete resolution over four weeks. Bell's palsy is still an uncommon potential side effect even though the advantages of the Hepatitis B vaccination exceed the risks. Further investigation is necessary to prove a conclusive link between the vaccine and its neurological side effects.

**Summary**

- Bell's palsy can occur as a rare neurological complication following the hepatitis B vaccine.
- While the condition is generally self‐limiting with a favorable prognosis, awareness of its association with vaccination is important for early diagnosis and treatment to ensure complete recovery.

## 1 Introduction

Bell's palsy is the most common cause of lower motor neuron paralysis and is characterized by acute‐onset unilateral peripheral facial neuropathy [1]. The pathogenesis of Bell's palsy is attributed to immune, infective, and ischemic mechanisms, but the exact cause remains unclear. The incidence of Bell's palsy in the United States ranges from 11.5 to 40.2 cases per 100,000 population [2]. The symptoms usually peak in 72 h to 1 week and gradually resolve over 3 weeks to 3 months [3]. This case report is according to CARE guidelines [4]. We present a case of Bell's palsy following the administration of the hepatitis B vaccine by a healthcare professional.

## 2 Case History/Examination

A 35‐year‐old female doctor presented to the neurology outpatient clinic with left‐sided facial weakness 6 days after administration of the recombinant hepatitis B vaccination. She started having complaints of heaviness over the left side of her face and neck, which progressed to weakness of both the upper and lower half of the left side of her face. It was not associated with any complaint of fever, upper respiratory symptoms, skin rash, ear vesicles or discharge, facial twitching or spasms, or systemic features. There was no history of recent travel. She had no localized pain, erythema, or swelling at the vaccination site. She was generally fit and healthy without any significant past medical history. She was not using any regular medications and did not report any allergies.

On examination, a young female without any obvious facial or head asymmetry had normal vitals and blood sugar levels of 86 mg/dL. On the left half of the face, there was loss of wrinkling with widened palpebral fissure, sagging of lower lid, sagging of eyebrow, inability to close the eye, flattening of the nasolabial fold, and drooping of the angle of the mouth. There was no involvement of other cranial nerves, and the rest of the neurological examination was normal. The parotid gland was not enlarged, and there were no palpable facial or cervical lymph nodes. There were no vesicles or discharge in the ear canal. The otoscopic examination was unremarkable. The injection site over the left deltoid showed no erythema tenderness or signs of infection.

## 3 Methods

On investigation, routine hematological workup (complete blood count, liver function tests, kidney function tests, serum electrolytes, blood sugar), inflammatory markers (ESR, CRP), chest X‐ray, and urine routine analysis were normal. Blood serology for HSV, EBV, and CMV was negative. MRI of the brain did not reveal any intracranial pathology. Extractable nuclear antigen (ENA) profile was also normal.

Initial differentials included viral infections (e.g., herpes simplex virus, varicella zoster virus), autoimmune conditions (e.g., multiple sclerosis, Guillain–Barré syndrome), diabetes mellitus, Lyme disease, trauma or injury to the facial nerve, tumors affecting the facial nerve, stroke, hypertension, ear infections or otitis media, sarcoidosis were ruled out based on clinical, and investigational data. Thus, a diagnosis of lower motor neuron type facial nerve palsy was made, and it was assumed most probably to be a sequel of vaccination against Hepatitis B.

The patient was given prednisolone 1 mg/kg/day or 7 days and pregabalin 75 mg at night for 2 weeks. Vitamin B complex was also given for 2 weeks. An ophthalmological evaluation was advised, along with artificial tears and an eye patch. After the 5th day, she was referred to a physiotherapist for nerve stimulation and routine exercises. The patient began to improve after 7 days, and facial weakness completely resolved 4 weeks after the onset of the illness.

At 6‐month and 1‐year follow‐up, the patient remained consistently asymptomatic.

## 4 Discussion

Vaccination against hepatitis B is crucial to reducing the incidence of hepatitis B virus‐associated infection. The hepatitis B vaccine has a favorable safety profile with a low incidence of adverse effects. The recombinant hepatitis B vaccine has excellent immunogenicity and protective efficacy in healthy individuals, with high seroprotection rates and tolerability similar to those of other hepatitis B vaccines [5].

However, some neurological adverse effects, though rare, have been attributed to the hepatitis B vaccine over the past decades. Lower motor neuron facial paralysis causing Bell's palsy is one such rare adverse effect. Facial paralysis can range from mild impairment to complete loss of movement. The severity of facial nerve dysfunction can be assessed using the House–Brackmann grading system as shown in Table 1 [6].

**TABLE 1:** House‐Brackmann facial nerve grading system.

House–Brackmann facial nerve grading system
Grade I—Normal Normal facial function in all areas
Grade II—Slight dysfunction Gross: slight weakness noticeable on close inspection; may have very slight synkinesis At rest: normal symmetry and tone Motion: forehead—moderate to good function; eye—complete closure with minimum effort; mouth—slight asymmetry
Grade III—Moderate dysfunction Gross: obvious but not disfiguring difference between two sides; noticeable but not severe synkinesis, contracture, and/or hemi‐facial spasm At rest: normal symmetry and tone Motion: forehead—slight to moderate movement; eye—complete closure with effort; mouth—slightly weak with maximum effort
Grade IV—Moderate severe dysfunction Gross: obvious weakness and/or disfiguring asymmetry At rest: normal symmetry and tone Motion: forehead—none; eye—incomplete closure; mouth—asymmetric with maximum effort
Grade V—Severe dysfunction Gross: only barely perceptible motion At rest: asymmetry Motion: forehead—none; eye—incomplete closure; mouth—slight movement
Grade VI—Total paralysis No movement

According to a pharmacovigilance study conducted by Jeong et al. in 2023, among 49,537 cases of all‐cause facial paralysis reported from 1967 to 2023, 26,197 were sought to be vaccine‐associated. Jeong et al. claimed a total of 495 cases of Bell's palsy to have been reported after the administration of the hepatitis B vaccine [7].

Bell's palsy is an exclusion diagnosis. Before a diagnosis of Bell's palsy is made, more serious congenital and acquired causes of LMN facial nerve palsy, including the infranuclear causes—Lyme disease, otitis media, Ramsay Hunt syndrome, sarcoidosis, Guillain–Barré syndrome, and tumor—and the supranuclear causes—multiple sclerosis, stroke, and tumor—need to be ruled out [8].

Alp et al. presented a case similar to ours; a 2‐year‐old girl presented with Bell's palsy 6 days after administration of the recombinant hepatitis B vaccine. No treatment was given, and the condition completely resolved 90 days after the initial presentation [9].

Grewal et al. presented a case of lower motor neuron palsy after administration of the hepatitis B vaccine, with all other possible causes ruled out. The case report highlights the development of abducens nerve palsy in a neonate 2 days after the administration of HBV. The patient recovered fully, with no recurrence of abducens nerve palsy, despite receiving the full HBV course and other recommended immunizations [10].

The exact etiology of Bell's palsy following the hepatitis B vaccine is unknown. It is postulated that latent Herpes simplex virus reactivation in the geniculate ganglion of facial nerves may be one of the causes of Bell's palsy. Some studies hypothesize that immune‐mediated demyelination may be implicated in the pathogenesis of Bell's palsy as it is associated with the development of Guillain–Barre syndrome after hepatitis B vaccine administration [8]. Therefore, at least a theoretical possibility of the development of Bell's palsy as a side effect of the recombinant hepatitis B vaccine is present. However, the exact cause and mechanism remain unknown.

Bell's palsy is a relatively benign condition with a favorable prognosis in most cases. Age, hypertension, and initial House–Brackmann grade are important prognostic factors [11]. However, clinicians should be able to differentiate it from other serious conditions to ensure appropriate management of patients with facial palsy.

## 5 Conclusion

Considering the risks versus benefits of the hepatitis B vaccine, the benefits certainly outweigh the risks. We propose that more studies be done to see if there is any evidence of neurological adverse effects associated with the vaccine. Until then, the global vaccination program should continue to eradicate the deadly virus.

## Author Contributions

**Raza Gulzar Ghouri:** conceptualization, writing – original draft. **Hamza Naeem:** writing – review and editing. **Mirza Rehan Yousaf:** writing – original draft. **Anam Sohail:** resources. **Waqas Arshad:** supervision. **Abdul Mabood Basil:** resources.

## Consent

Written informed consent has been obtained from the patient.

## Conflicts of Interest

The authors declare no conflicts of interest.

## Data Availability

Data is available upon request.
